# Risk of breast cancer and other cancers in heterozygotes for ataxia-telangiectasia

**DOI:** 10.1038/sj.bjc.6690209

**Published:** 1999-03

**Authors:** H M Inskip, L J Kinlen, A M R Taylor, C G Woods, C F Arlett

**Affiliations:** MRC Environmental Epidemiology Unit, University of Southampton, Southampton General Hospital, Southampton, SO16 6YD, UK; CRC Cancer Epidemiology Research Group, University of Oxford, The Radcliffe Infirmary, Oxford, OX2 6HE, UK; CRC Institute for Cancer Studies, The University of Birmingham, Edgbaston, Birmingham, B15 2TA, UK; Yorkshire Regional Genetics Service, Ashley Wing, Beckett Street, Leeds, LS9 7TF, UK; MRC Cell Mutation Unit, University of Sussex, Falmer, Brighton, BN1 9RR, UK

**Keywords:** ataxia-telangiectasia, breast cancer, cancer mortality

## Abstract

Mortality from cancer among 178 parents and 236 grandparents of 95 British patients with ataxia-telangiectasia was examined. For neither parents nor grandparents was mortality from all causes or from cancer appreciably elevated over that of the national population. Among mothers, three deaths from breast cancer gave rise to a standardized mortality ratio of 3.37 (95% confidence interval (CI): 0.69–9.84). In contrast, there was no excess of breast cancer in grandmothers, the standardized mortality ratio being 0.89 (95% CI: 0.18–2.59), based on three deaths. This is the largest study of families of ataxia-telangiectasia patients conducted in Britain but, nonetheless, the study is small and CIs are wide. However, taken together with data from other countries, an increased risk of breast cancer among female heterozygotes is still apparent, though lower than previously thought. © 1999 Cancer Research Campaign


*British Journal of Cancer* (1999) **79**(7/8), 1304–1307
© 1999 Cancer Research Campaign
Article no. bjoc.1998.0209

### Risk of breast cancer and other cancers in heterozygotes for ataxia-telangiectasia

**HM Inskip[1], LJ Kinlen[2], AMR Taylor[3], CG Woods[4] and CF Arlett[5]**

[1]MRC Environmental Epidemiology Unit, University of Southampton, Southampton General Hospital, Southampton SO16 6YD, UK; [2]CRC Cancer Epidemiology Research Group, University of Oxford, The Radcliffe Infirmary, Oxford OX2 6HE, UK; [3]CRC Institute for Cancer Studies, The University of Birmingham, Clinical Research Block, Edgbaston, Birmingham B15 2TA, UK; [4]Yorkshire Regional Genetics Service, Ashley Wing, St James's University Hospital, Beckett Street, Leeds LS9 7TF, UK; [5]MRC Cell Mutation Unit, University of Sussex, Falmer Brighton BN1 9RR, UK

**Summary** Mortality from cancer among 178 parents and 236 grandparents of 95 British patients with ataxia-telangiectasia was examined. For neither parents nor grandparents was mortality from all causes or from cancer appreciably elevated over that of the national population. Among mothers, three deaths from breast cancer gave rise to a standardized mortality ratio of 3.37 (95% confidence interval (CI): 0.69–9.84). In contrast, there was no excess of breast cancer in grandmothers, the standardized mortality ratio being 0.89 (95% CI: 0.18–2.59), based on three deaths. This is the largest study of families of ataxia-telangiectasia patients conducted in Britain but, nonetheless, the study is small and CIs are wide. However, taken together with data from other countries, an increased risk of breast cancer among female heterozygotes is still apparent, though lower than previously thought.

**Keywords:** ataxia-telangiectasia; breast cancer; cancer mortality

Elevated risks of cancers, particularly lymphoma and leukaemia, have been well documented in sufferers of the autosomal recessive disorder ataxia-telangiectasia (A-T). Homozygotes are rare with estimates being of the order of 1 per 100 000 births. Heterozygote carriers of the A-T gene are more common but remain free of symptoms and are usually only identified when they produce a child with A-T. Recently, however, the gene, *ATM*, which is mutated in A-T patients, has been identified (Savitsky et al, 1995) and this has led to the possibility of screening for carriers. Because heterozygotes have in the past only been identified through their offspring, the ability to study such individuals has been limited by their small numbers. Despite this, however, an increased incidence of cancer, and in particular of breast cancer, has been reported from the USA (Swift et al, 1987, 1991) and an increase in breast cancer has been observed in Norway, albeit based on a very small numbers of cases (Borrensen et al, 1990). A study in Britain (Pippard et al, 1988) found an excess of breast cancers in mothers but not in grandmothers of A-T patients, but again it involved only a small number of subjects.

Given that A-T heterozygotes constitute 0.2–1% of the population, they may contribute significantly to the population burden of breast cancer. Screening for the mutated A-T gene is a difficult procedure, and thus it is important to establish the risk of breast cancer in heterozygotes so that the effectiveness of genetic screening can be determined. To this end, we have combined data from the only two sources of data in the UK containing follow-up information on parents and grandparents of A-T cases to estimate the risk of breast cancer in UK heterozygotes.

*Received 30 July 1998*
*Accepted 1 October 1998*

*Correspondence to:* HM Inskip

### MATERIALS AND METHODS

The A-T patients whose relatives have been followed up derive from two British series. The first consists of the group previously studied in Southampton by Pippard and her colleagues (1988). The methods were described in detail in the earlier paper, but, briefly, the A-T cases were ascertained by contacting all consultant dermatologists, paediatricians and neurologists in Britain in 1985. They were asked to provide details of A-T cases known to them who had been diagnosed since 1945. After permission was obtained from the patients' consultants and general practitioners (GPs), the patients' families were contacted and identifying details for the parents and grandparents were requested. The cases, parents and grandparents were then traced and flagged in the National Health Service Central Register (NHSCR) so that information could be obtained on emigrations, deaths and cancer registrations that occurred in the study population.

The second group of A-T patients, assembled by LJK in Oxford, comprised a clinical series studied by DG Harnden together with additional cases with A-T certified as the cause of death. These deaths were identified from an examination of all death certificates for England and Wales for the years 1959–1978 in which the underlying cause of death bore the codes that embrace A-T. So far as was possible, identifying particulars were obtained from birth certificates and Family Practitioner Committees for parents and grandparents to be traced and flagged in the NHSCR. No contact was made with the families.

The study of Pippard et al (1988) was taken as the primary source, so those families that were duplicated in the Oxford series were eliminated from the present study. No new families have been added to these original series so they do not contain data on families of A-T cases diagnosed after 1985.

As described in the first paper, the date of entry of an A-T mother was the date of birth of the proband; that of a grandmother, the birth of her child who subsequently became the mother of a

**Table 1** Analysis of parents. Numbers of observed deaths (Obs), SMRs and 95% CIs for selected causes of death

| | Fathers | | | Mothers | | | All parents | | |
|---|---|---|---|---|---|---|---|---|---|
| **Cause (ICD 9th revision codes)** | **Obs** | **SMR** | **95% CI** | **Obs** | **SMR** | **95% CI** | **Obs** | **SMR** | **95% CI** |
| All malignant neoplasms (140–208) | 7 | 1.53 | 0.61–3.15 | 4 | 1.26 | 0.34–3.21 | 11 | 1.42 | 0.71–2.53 |
| Cancer of: | | | | | | | | | |
| Stomach (151) | 0 | 0 | 0–9.82 | 1 | 8.39 | 0.21–46.74 | 1 | 2.02 | 0.05–11.26 |
| Colon (153) | 0 | 0 | 0–12.30 | 0 | 0 | 0–17.22 | 0 | 0 | 0–7.17 |
| Rectum (154) | 1 | 4.90 | 0.12–27.30 | 0 | 0 | 0–395.84 | 1 | 3.36 | 0.09–18.74 |
| Lung (162–164) | 5 | 2.96 | 0.96–6.90 | 0 | 0 | 0–8.18 | 5 | 2.34 | 0.76–5.45 |
| Female breast (174)[a] | – | – | – | 3 | 3.37 | 0.69–9.84 | 3 | 3.37 | 0.69–9.84 |
| Kidney (191–192) | 1 | 9.24 | 0.23–51.48 | 0 | 0 | 0–87.07 | 1 | 6.64 | 0.17–37.00 |
| All causes (000–999) | 17 | 1.08 | 0.63–1.73 | 7 | 0.89 | 0.36–1.83 | 24 | 1.02 | 0.65–1.51 |

[a]For age groups < 40, 40–49, 50–59 and ≥ 60 years, the observed numbers of deaths from female breast cancer were 1, 0, 1 and 1, respectively and the expected deaths were 0.09, 0.28, 0.31 and 0.21. Information on specific cancer sites is presented for those for which there was at least one observed death or the expected number of deaths exceeded 0.5.

**Table 2** Analysis of grandparents. Numbers of observed deaths (Obs), SMRs and 95% CIs for selected causes of death

| | Grandfathers | | | Grandmothers | | | All grandparents | | |
|---|---|---|---|---|---|---|---|---|---|
| **Cause (ICD 9th revision codes)** | **Obs** | **SMR** | **95% CI** | **Obs** | **SMR** | **95% CI** | **Obs** | **SMR** | **95% CI** |
| All malignant neoplasms (140–208) | 26 | 1.24 | 0.81–1.82 | 20 | 1.22 | 0.74–1.88 | 46 | 1.23 | 0.90–1.64 |
| Cancer of: | | | | | | | | | |
| Oesophagus (150) | 1 | 1.59 | 0.04–8.84 | 1 | 2.35 | 0.06–13.07 | 2 | 1.89 | 0.23–6.84 |
| Stomach (151) | 4 | 1.75 | 0.48–4.47 | 2 | 1.64 | 0.20–5.93 | 6 | 1.71 | 0.63–3.72 |
| Colon (153) | 0 | 0 | 0–2.62 | 3 | 1.90 | 0.39–5.56 | 3 | 1.00 | 0.21–2.93 |
| Rectum (154) | 2 | 1.96 | 0.24–7.06 | 0 | 0 | 0–5.23 | 2 | 1.16 | 0.14–4.18 |
| Pancreas (157) | 1 | 1.16 | 0.03–6.46 | 2 | 2.77 | 0.34–9.99 | 3 | 1.89 | 0.39–5.53 |
| Lung (162–164) | 9 | 1.13 | 0.52–2.15 | 1 | 0.44 | 0.01–2.46 | 10 | 0.98 | 0.47–1.80 |
| Female breast (174)[a] | | | | 3 | 0.89 | 0.18–2.59 | 3 | 0.89 | 0.18–2.59 |
| Cervix uteri (180) | | | | 2 | 2.94 | 0.36–10.63 | 2 | 2.94 | 0.36–10.63 |
| Ovary (183) | | | | 1 | 0.90 | 0.02–5.00 | 1 | 0.90 | 0.02–5.00 |
| Prostate (185) | 0 | 0 | 0–2.08 | | | | 0 | 0 | 0–2.08 |
| Bladder (188) | 2 | 2.11 | 0.26–7.64 | 0 | 0 | 0–11.06 | 2 | 1.56 | 0.19–5.65 |
| Kidney etc. (189) | 0 | 0 | 0–11.26 | 0 | 0 | 0–18.62 | 0 | 0 | 0–7.02 |
| Brain (191, 192) | 0 | 0 | 0–11.86 | 0 | 0 | 0–13.59 | 0 | 0 | 0–6.33 |
| Non-Hodgkin's lymphoma (200, 202.0, 202.1, 202.8) | 1 | 3.25 | 0.08–18.10 | 0 | 0 | 0–12.65 | 1 | 1.67 | 0.04–9.29 |
| Leukaemia (204–208) | 0 | 0 | 0–8.03 | 0 | 0 | 0–9.98 | 0 | 0 | 0–4.45 |
| All causes | 85 | 0.94 | 0.75–1.17 | 71 | 112 | 0.87–1.40 | 158 | 1.02 | 0.86–1.19 |

[a]For age groups < 40, 40–49, 50–59 and ≥ 60 years, the observed numbers of deaths from female breast cancer were 0, 0, 1 and 2, respectively, and the expected deaths were 0.06, 0.36, 0.79 and 2.19. Information on specific cancer sites is presented for those for which there were at least two observed deaths or the expected number of deaths exceeded 0.5.

specific case; that of fathers and grandfathers, the dates of conception (instead of dates of birth) of their offspring. The starting date of the study was set at 1 January 1951 because of the difficulty of tracing prior to this date of entry for some individuals. Follow-up continued to the end of 1994.

Standard methods of analysis were used to compare the observed numbers of deaths from all causes, all cancers and from specific cancers with those expected from national rates (Breslow and Day, 1987). For each sex, person-years at risk in 5-year age groups and 5-year calendar periods were derived; expected numbers of deaths were then obtained by multiplying the person-years by the corresponding national mortality rates, and then summing across relevant categories. Standardized mortality ratios (SMRs) were calculated as the ratio of observed to expected deaths. The 95% confidence intervals (CIs) were derived from tables of the Poisson distribution. Separate analyses were performed for parents and grandparents.

### RESULTS

Follow-up data were available for at least one parent or grandparent from each of 57 families from the Southampton study and an additional 44 families from the Oxford study. However, six of the latter had to be excluded as they were already in the Southampton set.

Information for 37 families in the combined dataset was complete in that data were available for both parents and all four grandparents. In total, information on 178 parents and 240 grandparents from 95 families was available for the analysis, but four of

© Cancer Research Campaign 1999

**Table 3** Estimated risks for breast cancer in A-T heterozygotes (adapted from Easton 1994)

| Study type | Reference No. | Study | Breast cancer Cases | RR | 95% CI | Other cancers Cases | RR | 95% CI |
|---|---|---|---|---|---|---|---|---|
| Epidemiological | 1 | Swift et al (1987) | 27 | 6.8 | 2.0–22.6 | 67[a] | 2.3 | 1.3–3.9 |
| | | | | | | 44[b] | 2.9 | 1.5–5.5 |
| Epidemiological | 2 | Swift et al (1991) | 23 | 5.1 | 1.5–16.9 | 68 | 3.5 | 2.0–6.1 |
| Epidemiological | 3 | Borresen et al (1990) | 6 | 3.9 | 1.3–12.1 | 14 | 0.8 | 0.3–1.9 |
| Epidemiological | 4 | Present study | 6 | 1.7 | 0.6–4.3 | 51 | 1.4 | 1.0–2.0 |
| Mutational analysis | 5 | Athma et al (1996)[c] | 25 | 3.8 | 1.7–8.4 | | | |
| Mutational analysis | 6 | Fitzgerald et al (1997) | 2 | 0.5 | 0.04–7.0 | | | |
| Both | 3–6 | Total | 39 | 2.7 | 1.6–4.6 | | | |
| Both | 2, 3, 4, 6 | Total[d] | 37 | 2.6 | 1.4–4.8 | | | |
| Epidemiological | 1–4 | Total | 62 | 3.5 | 2.0–6.0 | 244 | 1.9 | 1.5–2.4 |

[a]Males. [b]Females. [c]This study appears to comprise cases from studies 1 and 2. [d]Total for studies excluding any cases from study 1 as this study was the source of the breast cancer hypothesis.

the grandparents died before 1 January 1951 and so were excluded. Parents were followed up for an average of 27 years and grandparents for 32 years. As would be expected from their ages, more deaths were observed among grandparents than parents. Numbers of deaths and standardized mortality ratios for these two groups are provided in Tables 1 (parents) and 2 (grandparents).

Among parents, there were only 11 deaths from cancer, but no appreciable excess (SMR 1.42; 95% CI 0.71–2.53) as shown in Table 1. Among mothers, three deaths from breast cancer were recorded, giving a SMR of 3.37 (95% CI 0.69–9.84). There was also no appreciable increase of deaths from cancer among grandparents (observed 46, SMR 1.23; 95% CI 0.90–1.64). In the case of breast cancer, three deaths were observed among grandmothers (SMR 0.89, 95% CI 0.18–2.59). An examination of deaths from all causes similarly provided no remarkable findings in either parents or grandparents (Tables 1 and 2).

Two additional breast cancers were notified to us, one in a mother and the other in a grandmother. These cases were, however, still alive at the end of 1994. Of the six deaths from breast cancer, only three were notified to us as cancer registrations. Further analysis of cancer registration data has not been performed, mainly because cancer incidence data are only considered reliable for more recent years.

The numbers of deaths from other specific cancers were small, though amongst those cancer sites with more than five deaths, there were suggestions of excesses of lung cancer in fathers and stomach cancer in grandparents, but with wide CIs. The number of deaths from lung cancer in grandparents almost exactly equalled the number expected on the basis of national rates, giving an SMR of 0.98.

### DISCUSSION

In this study, the available information on tracing of A-T heterozygotes in Britain has been drawn together and updated. The original Southampton study (Pippard et al, 1988) reporting on follow-up until mid-1986 found an SMR of 11.87 (based on two deaths) for breast cancer in mothers of A-T patients. No breast cancers were reported in grandmothers, of whom half would be expected to be heterozygotes. Easton (1994) reported an analysis of the same study with follow-up to the end of 1992, by which time there had been no further breast cancer cases. With the extended follow-up, the expected number of cases increased and the SMR fell to 6.31. The addition of the data from Oxford has considerably increased the person-years at risk and the expected deaths from breast cancer have increased threefold. In contrast, the observed number of cases has only increased by the addition of one from the Oxford data. No further cases of breast cancer were observed in the Southampton study. The combined SMR of 3.37 (95% CI 0.69–9.84) in mothers is therefore the lowest yet reported from Britain, but it is based on a larger dataset than available before. However, the study numbers are still small and thus the CI is wide.

Considering mothers and grandparents together, six deaths from breast cancer were observed, compared to 4.28 expected. If the expected number is adjusted to allow for the fact that only 50% of grandparents are heterozygotes, the estimated relative risk (RR) becomes 1.66 (95% CI 0.65–4.28).

These findings are compared in Table 3 with those from other studies, following the approach used by Easton (1994). The present study finds a much lower risk of breast cancer than either the US or the Norwegian follow-up studies (Swift et al, 1987; 1991; Borreson et al, 1990). This is mainly because of the lack of any increase among grandmothers. The difference is intriguing and may be due to chance. It has also been suggested that it reflects a susceptibility to an environmental factor that was unimportant in the grandmother generation (Bridges and Arlett, 1992). Another explanation is that we may have failed to trace a few grandmothers in the NHSCR because they died from breast cancer before the start of the study. We have no direct evidence of this, but the fact that identifying details were easier to obtain for relatives who were living at the time the A-T case was ascertained (mainly in the early 1980s) must make this a possibility. In this connection, it may be relevant to note that, whereas among parents there was an indication of an excess of lung cancer, which is well known as being related to social class, this was not found among the grandparents. This might reflect again an underascertainment of grandparents because of an earlier excess mortality from certain causes.

Now that the *ATM* gene has been identified (Savitsky et al, 1995), there have been other approaches to examining the risk of breast cancer in heterozygotes. Recently, Athma et al (1996) have genotyped 775 relatives in 99 A-T families, including 33 women with breast cancer. From this they were able to derive an estimated

 © Cancer Research Campaign 1999

odds ratio (OR) of 3.8 (95% CI 1.7–8.4) for the risk of breast cancer in heterozygotes. In contrast, however, Fitzgerald et al (1997) found no increased risk of breast cancer in young heterozygote women. They performed a germ-line mutational analysis of 401 women with breast cancer diagnosed at under age 40 and compared them with similar analyses of 202 controls. Mutations of the A-T gene were found in 0.5% of the cases and 1% of the controls, leading to an estimated OR of 0.5 (95% CI 0.04–6.97). Bishop and Hopper (1997) have, however, noted that these studies are not incompatible and argue that further detailed and large-scale population-based studies are needed before we can be clear about the risk in heterozygotes. These two genetic studies have been added to Table 3 and similar summary RRs are obtained independently of whether the hypothesis-generating study of Swift et al (1987) is included or not. These point to an overall increase in risk of breast cancer of approximately two- to three-fold in heterozygotes but with a wide CI.

Although the findings of available studies point to an increased risk of breast cancer among A-T heterozygotes, there continues to be a need for additional data. This particularly applies to the assessment of whether the risk varies with age. There are suggestions that younger women are at greater risk; Swift et al (1987) noted that two of the four breast cancers in mothers in their study were at unusually young ages, and one of the mothers in the present study died from this malignancy at age 38 years having been diagnosed at the age of 34. In contrast, however, the genetic study by Athma et al (1996) found the risk to be greatest in those over 60 years old.

In addition to the concern about breast cancer, the possible importance of the *ATM* gene in other sporadic tumours has recently been highlighted by reports of *ATM* mutations in T-cell prolymphocytic leukaemia (T-PLL) in non-A-T patients (Stilgenbauer et al, 1997; Vorechovsky et al, 1997). Although *ATM* mutations in some cases of sporadic T-PLL have been shown to be acquired (Stoppa-Lyonnet et al, 1998), and no sporadic T-PLL case has so far been shown to carry a germline *ATM* mutation, the risk of T-PLL in A-T heterozygotes remains unknown.

The present study has added to the information available about the cancer risk in heterozygotes. It pools the only available information on follow-up of such individuals in the UK, but despite this the numbers are small. However, the findings taken alongside those from studies elsewhere do indicate an elevated risk of breast cancer in female heterozygotes though its true magnitude is still uncertain.

### ACKNOWLEDGEMENTS

We are grateful for the help provided by Bryn Bridges, Jane Cole, Douglas Easton, Shirley Simmonds, Paul Winter and the staff of the National Health Service Central Register. We are also indebted to the A-T patients and their families and physicians who provided information for this study.

### REFERENCES

Athma P, Rappaport R and Swift M (1996) Molecular genotyping shows that ataxia-telangiectasia heterozygotes are predisposed to breast cancer. *Cancer Genet Cytogenet* **92**: 130–134

Bishop DT and Hopper J (1997) AT-tributable risks? *Nat Genet* **15**: 226

Borresen AL, Anderson TL, Treti S, Heiberg A and Moller P (1990) Breast cancer and other cancers in Norwegian families with ataxia-telangiectasia. *Genes Chromosom Cancer* **2**: 339–340

Breslow NE and Day NE (1987) *Statistical Methods in Cancer Research, Vol 2. The Design and Analysis of Cohort Studies*. International Agency for Research on Cancer Lyon

Bridges B and Arlett C (1992) Risk of breast cancer in ataxia-telangiectasia. *N Engl J Med* **326**: 1357

Easton DF (1994) Cancer risks in A-T heterozygotes. *Int J Radiat Biol* **6**: S177–S182

Fitzgerald MG, Bean JM, Hegde SR, Unsal H, MacDonald DJ, Harkin DP, Finkelstein DM, Isselbacher KJ and Haber DA (1997) Heterozygous *ATM* mutations do not contribute to early onset of breast cancer. *Nat Genet* **15**: 307–310

Pippard EC, Hall AJ, Barker DJP and Bridges BA (1988) Cancer in homozygotes and heterozygotes of ataxia-telangiectasia and xeroderma pigmentosum in Britain. *Cancer Res* **48**: 2929–2932

Savitsky K, Bar-Shira A, Gilad S, Rotman G, Ziv Y, Vanagaite L, Tagle DA, Smith S, Uziel T, Sfez S, Ashkenazi M, Pecker I, Frydman M, Harnik R, Patanjali SR, Simmons A, Clines GA, Sartiel A, Gatti RA, Chessa L, Sanal O, Lavin MF, Jaspers NGJ, Taylor AMR, Arlett CF, Miki T, Weissman SM, Lovett M, Collins FS and Shiloh Y (1995) A single ataxia telangiectasia gene with a product similar to PI-3 kinase. *Science* **268**: 1749–1753

Stilgenbauer S, Schaffner C, Litterst A, Liebisch P, Gilad S, Bar-Shira A, James MR, Lichter P and Dohner H (1997) Biallelic mutations in the *ATM* gene in T-prolymphocytic leukaemia. *Nat Med* **3**: 1155–1160

Stoppa-Lyonnet D, Soulier J, Lauge A, Dastot H, Garand R, Sigaux F and Stern MH (1998) Inactivations of the ATM gene in T cell prolymphocytic leukaemia. *Blood* **91**: 3920–3926

Swift M, Reitnauer PJ, Morrell D and Chase CL (1987) Breast and other cancers in families with ataxia-telangiectasia. *N Engl J Med* **316**: 1289–1294

Swift M, Morrell D, Massey RB and Chase CL (1991) Incidence of cancer in 161 families affected by ataxia-telangiectasia. *N Eng J Med* **325**: 1831–1836

Vorechovsky I, Luo L, Dyer MJS, Catovsky D, Amlot P, Yaxley JC, Foroni L, Hammarstrom L, Webster ADB and Yuille MAR (1997) Clustering of missense mutations in the ataxia-telangiectasia gene in a sporadic T cell leukaemia. *Nat Genet* **17**: 96–99

© Cancer Research Campaign 1999
